# Clinical burden of dry eye disease in patients with ocular graft versus host disease

**DOI:** 10.20935/acadmedhealth8096

**Published:** 2026-01-09

**Authors:** Cherie B. Nau, Muriel M. Schornack, Jennifer S. Harthan, Jennifer S. Fogt, Amy C. Nau, William J. Hogan, Ellen S. Shorter

**Affiliations:** 1 Department of Ophthalmology, Mayo Clinic, Rochester, MN, USA.; 2 Illinois College of Optometry, Chicago, IL, USA.; 3 College of Optometry, The Ohio State University, Columbus, OH, USA.; 4 Forefront Eye Care, Boston, MA, USA.; 5 Division of Hematology, Mayo Clinic, Rochester, MN, USA; 6 Department of Ophthalmology and Visual Sciences, University of Illinois at Chicago, Chicago, IL, USA.

**Keywords:** dry eye disease, meibomian gland dysfunction, ocular graft-versus-host disease, scleral contact lens, stem cell transplant

## Abstract

**Background:**

Ocular graft-versus-host disease (oGVHD) is a frequent complication after hematopoietic stem cell transplant, with dry eye being the most common presentation. Patient experience with dry eye from oGVHD may negatively affect quality of life. The purpose of this study was to evaluate patients’ perspectives on the symptoms, treatments, and care burdens associated with dry eye from oGVHD.

**Materials and methods:**

An electronic questionnaire was sent to patients with dry eye asking about their diagnoses, symptoms, treatments, and experiences associated with dry eye.

**Results:**

Of the 639 patients who responded to the questionnaire, 79 reported having dry eye from oGVHD and were included in the analysis. The mean (SD) age of these patients was 55 (10) years (range, 22–73 years; *n* = 75). Of the patients who reported their sex (*n* = 78), 32 were male, and 46 were female. The most common ocular symptom was a sandy or gritty feeling (*n* = 68, 86%). Patients reported a mean (SD) of nine (2) symptoms, despite using four (2) treatments. The most common initial treatment was artificial tears (*n* = 71, 90%). Most patients (68/75, 91%) reported receiving oGVHD care from an eye care professional (e.g., ophthalmologist or optometrist), whereas a few (4/75, 5%) received oGVHD care from a hematologist.

**Conclusions:**

Dry eye symptoms and treatments, along with the associated time and financial costs required, are a burden to patients with oGVHD. These patients should be evaluated by a knowledgeable eye care professional to best treat and manage their disease.

## 1. Introduction

Ocular graft-versus-host disease (oGVHD) occurs in 41% to 60% of patients who undergo allogeneic hematopoietic stem cell transplant (HSCT) [1]. The most common manifestation of oGVHD is severe keratoconjunctivitis [2]. The inflammatory sequelae of oGVHD affect the cornea, conjunctiva, lacrimal gland, meibomian glands, and eyelids and result in irreversible fibrosis and diminished tissue function [3–7]. Other ocular tissues may also be affected by oGVHD, although less often. oGVHD symptoms may develop at any time after transplant. One study in which 111 patients were evaluated after HSCT for 3 years showed that the signs and symptoms of dry eye were increased during the second year after HSCT. In a study of 53 patients with dry eye, the mean (SD) time to diagnosis of dry eye after HSCT was 171 (59) days [8].

According to guidelines published in 2014, distinctive features of oGVHD are the new onset of dry, gritty, or painful eyes; cicatricial conjunctivitis; keratoconjunctivitis sicca; or confluent areas of corneal epithelial damage. Other features include photophobia, periorbital hyperpigmentation, and blepharitis [9], although the list of symptoms is largely subjective. These guidelines suggest staging eye involvement according to how a patient’s symptoms affect their daily activities and how often lubricant drops are used during the day. A formal evaluation by an eye care professional is recommended. The International Chronic Ocular GVHD Consensus Group created a clinical severity scale to grade oGVHD with objective findings of Schirmer testing, corneal fluorescein staining, conjunctival injection, and objective measure of the Ocular Surface Disease Index questionnaire [10].

Inflammatory damage to lacrimal and meibomian glands results in severe dry eye disease. The lacrimal glands produce the aqueous component of tears, which maintains corneal epithelial integrity through continuous hydration. oGVHD-related inflammation can cause irreversible atrophy of the lacrimal gland. Meibomian glands, located in the upper and lower eyelids, produce meibum, which forms the outer lipid layer of the tear film. This lipid layer prevents evaporation of the underlying aqueous component and stabilizes the tear film on the ocular surface. The tear film is critically important for protecting the cornea and providing a smooth optical surface for clear vision. Therefore, disruption to the tear film due to oGVHD can result in debilitating symptoms, persistent epithelial defects, loss of corneal sensation, and corneal perforation [7,11].

Few studies have reported patients’ experiences with oGVHD. In a study of patients with graft-versus-host disease (GVHD) after HCST, those with oGVHD scored worse on a quality-of-life assessment than patients without ocular symptoms [1]. On average, patients with oGVHD have 5 ocular symptoms and manage their dry eye with 3 treatments [12]. Patients with oGVHD not only experience ocular discomfort and reduced visual clarity but also may have higher treatment and financial burdens [13]. If oGVHD symptoms interfere with work productivity, the financial burden of treatment may have an even greater effect on quality of life [14,15].

Survival after HSCT has improved; therefore, health care professionals should be aware of the effect of persistent symptoms and treatments on patients’ quality of life. The purpose of this study was to expand our understanding of patients’ perspectives on symptoms, treatments, and care burdens associated with dry eye from oGVHD. We did not assess clinical signs of oGVHD but rather the personal experiences of patients with oGVHD without the influence of health care professionals.

## 2. Materials and methods

The institutional review board of the University of Illinois, Chicago, approved this study. Study data were collected and managed by using REDCap (Research Electronic Data Capture) hosted at the University of Illinois, Chicago. REDCap (version 7.3) is a secure, web-based software platform designed to support data capture for research studies [16,17]. As previously described, an electronic link to the questionnaire was shared with administrators of online support groups and foundations for patients with conditions associated with dry eye disease. The questionnaire was available from March through October 2018. A complete list of the groups contacted and questions asked is available elsewhere [18]. Data was collected on dry eye patients with any indication; however, this review consists of a sub-analysis of only those who self-reported oGVHD.

In addition, patients were asked to report their sex, age, and length of time since their initial dry eye diagnosis. Patients were asked to select their current ocular symptoms and initial and current treatments for dry eye disease from a list of symptoms and treatments provided. Patients also completed the Ocular Surface Disease Index (OSDI) questionnaire to evaluate their current state of dry eye disease [19]. The OSDI questionnaire consists of 12 questions to assess a patient’s dry eye symptoms during the previous week. A score of 0 to 23 is categorized as normal; 24 to 33, moderate dry eye; and 34 or greater, severe dry eye.

Additionally, patients were asked what type of specialist provided most of their eye care related to dry eye disease, whether they felt they were kept up to date with advances in dry eye therapy, whether it was easy to locate a specialist, the number of eye care visits per year, the amount of time spent per day on ocular care form a list of times provided, and to enter an estimated value for the yearly out-of-pocket expenses related to dry eye disease. Patients using contact lenses also reported the type of lenses used, the mean hours of daily wear, whether they experienced midday fogging or clouding of vision, and whether they needed to remove and reapply lenses during the day.

Not all questions were answered by each patient. Some questions may not have been applicable to each subject, or they may not have been comfortable to answer; thus, the number of patients that responded to each question is reported. The OSDI is scored based on the number of questions answered. Patients were not physically examined; therefore, visual acuity and clinical assessment of ocular surface integrity were not recorded. Only patients with dry eye who reported a diagnosis of oGVHD were included in the analysis. Data are reported by using descriptive statistics.

## 3. Results

Of the 639 patients with dry eye who responded to the questionnaire, 79 (12%) reported a diagnosis of oGVHD and were included in further analyses. The mean (SD) age of these patients was 55 (10) years (range, 22–73 years; *n* = 75). Of the 78 patients who reported their sex, 32 (41%) were male, and 46 (59%) were female. Patients reported being diagnosed with dry eye disease a median (IQR) of 4 (2–8) years (range, 0–31 years) before the questionnaire was completed (Table 1).

### 3.1. Symptoms

The 3 most common ocular symptoms reported by patients were a sandy or gritty feeling (*n* = 68, 86%), blurry vision (*n* = 64, 81%), and sensitivity to light (*n* = 63, 80%) (Table 2). Of the 11 ocular symptoms queried, patients reported a mean (SD) of 9 (2) current symptoms (range, 0–11). The complete list of symptoms is provided in Table 2. The mean (SD) OSDI score of the patients was 56 (23) (range, 10–100; *n* = 79), which falls under the severe dry eye category. Most patients (*n* = 66, 84%) scored in the severe dry eye range. There was not a strong correlation between the OSDI score and the number of symptoms, R^2^ = 26%.

### 3.2. Treatments

The three most common initial dry eye treatments were artificial tears (*n* = 71, 90%), eyelid warm compresses (*n* = 41, 52%), and punctal occlusion with a punctal plug (*n* = 37, 47%) (Table 3). Patients reported using a mean (SD) of 4 (2) (range, 1–9) current therapies for dry eye disease. The most frequently used initial treatments were consistent with the most commonly used current treatments (artificial tears, *n* = 69, 87%; warm compresses, *n* = 42, 53%; punctal occlusion, *n* = 30, 38%). Contact lenses were reported as a current treatment (*n* = 31; 39%) more frequently than as an initial treatment (*n* = 11; 14%). Of the patients who currently used contact lenses for treating dry eye disease, 81% (25/31) wore scleral lenses.

Problems with midday fogging or clouding of vision during lens wear were a common issue affecting 84% (21/25) of scleral lens wearers and 83% (5/6) of soft lens wearers.

### 3.3. Financial and time burden

**Figure 1.**
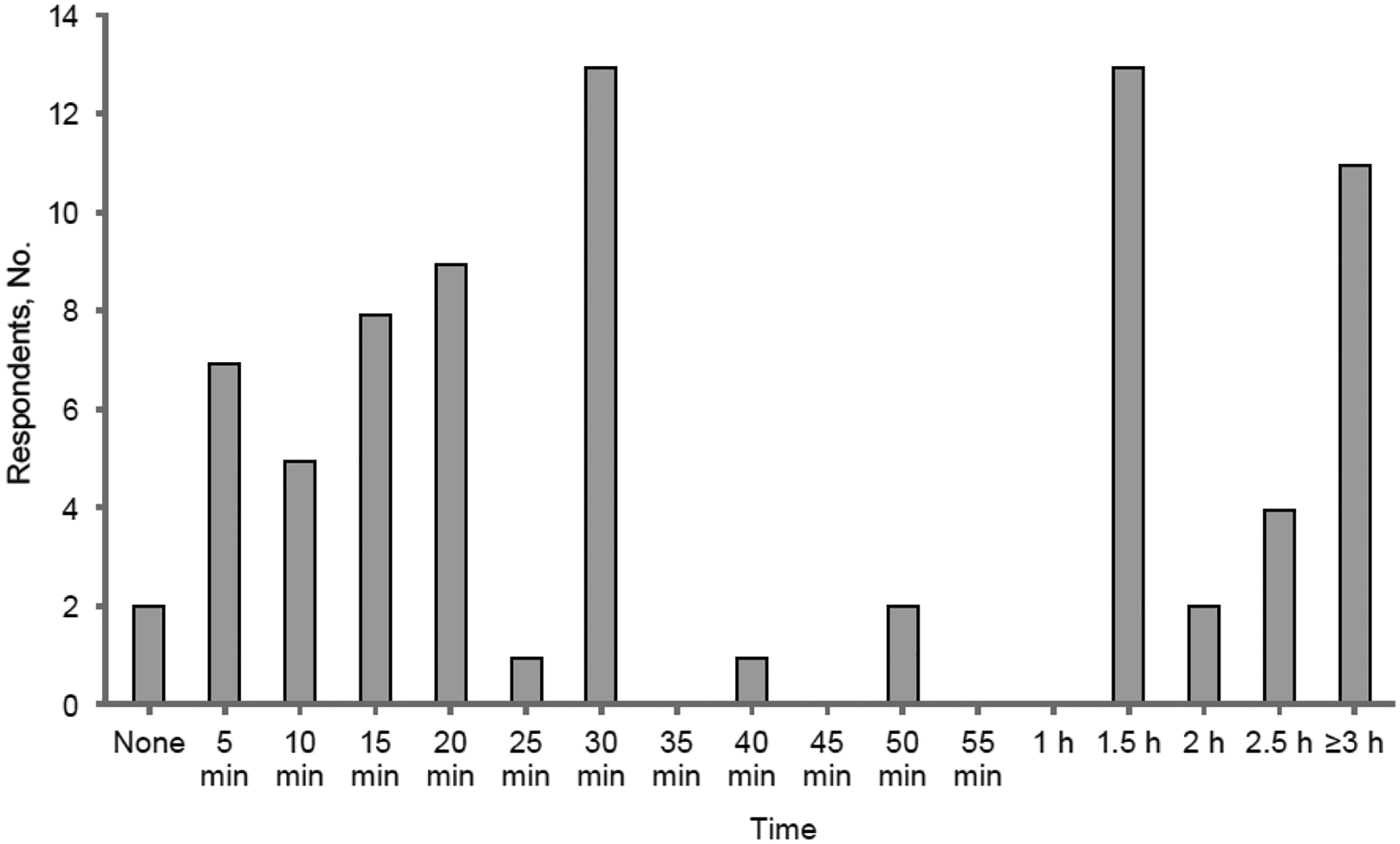
Daily time spent on eye care reported by patients (*n* = 78). Most patients (58%; *n* = 45) spent 30 min or less. However, 38% (*n* = 30) spent an hour or more a day on eye care.

The estimated annual median (IQR) out-of-pocket cost for ocular treatments was USD 900 (USD 400– USD 2000) (range, USD 0– USD 65,000; *n* = 74). Of 78 patients with oGVHD, 14 (18%) reported spending 10 or fewer minutes per day on ocular care; 31 (40%) reported spending 15 to 30 min per day on ocular care; and 30 (38%) reported spending an hour or more per day on ocular care (Figure 1).

### 3.4. Primary eye care professionals and number of examinations

Most patients reported that an ophthalmologist was their primary eye care professional (57/75, 76%), followed by an optometrist (11/75, 15%) and a hematologist (4/75, 5%). Although 74% (57/77) of patients thought their eye care professional was up to date on available dry eye treatments, only 40% (31/78) found it easy to locate an eye care professional specializing in dry eye. Patients attended a median (IQR) of 3.0 (2.0–4.5) (range, 0–20.0; *n* = 75) visits to their eye care professional each year.

## 4. Discussion

Despite using a mean (SD) of four (2) dry eye treatments, patients with oGVHD reported a mean (SD) of nine (2) current ocular symptoms. This slightly exceeded the findings of another study in which 193 patients with oGVHD were surveyed and reported a mean (SD) of three (3) treatments and five (2) residual symptoms, despite treatment [12]. In that study, 87% of patients reported a gritty feeling in the eyes, and 61% reported light sensitivity, compared with 87% of patients in our study who reported a sandy or gritty feeling in the eyes and 80% who reported light sensitivity [12]. Both studies showed a high percentage of patients with substantial ocular symptoms, despite multiple treatments. Regardless of a patient’s ocular examination findings, the symptoms and burden related to oGVHD may negatively affect the person’s daily experience. Although maintaining a healthy ocular surface is a top priority for eye care professionals, patient symptoms should be addressed.

In the current study, the mean OSDI score of patients indicated that their residual symptoms were severe (mean [SD] OSDI score, 56 [23]). This score is higher than the OSDI score of 31.1, representing moderate dry eye, that was reported in a 2014 study of 14 patients with oGVHD [20]. In that study, eight patients were using two or more treatments for dry eye. In another study of patients after HSCT, 28% (31/111) had OSDI scores greater than 20, and 33% (37/111) were using artificial tears for treatment [21]. The present study focused on patients with dry eye; 84% of the OSDI scores were in the severe dry eye category (i.e., OSDI score > 33), despite treatments. In a 2015 study of 84 patients with oGVHD, a mean (SD) OSDI score of 42.5 (24.1) was reported [22]. In addition, higher OSDI scores were significantly correlated with lower visual quality of life scores (National Eye Institute Visual Function Questionnaire [NEI-VFQ-25])[23].

The most common initial and current treatments identified in the current study were artificial tears (90% and 87%, respectively). This is similar to the findings of a previous study, in which 86% of patients reported the current use of artificial tears [7]. In the study of 111 patients after HSCT, the most common treatment was artificial tear use (33%), followed by topical corticosteroids (7%), although it should be noted that the study population included patients without a diagnosis of oGVHD [21]. In the current study, warm compresses were the second most common initial and current treatment (52% and 53%, respectively). The frequent use of warm compresses provides evidence that meibomian gland dysfunction is a common ocular complication of oGVHD [7,24]. However, warm compresses were not listed as a treatment in some studies [12,21]. In the current study, the greatest reduction from initial to current treatment was in the use of ophthalmic cyclosporine, with 44% of patients reporting cyclosporine as an initial treatment, and 22% reporting it as a current treatment. Ophthalmic cyclosporine may have been discontinued due to its cost, adverse effects, or ineffectiveness. The greatest increase in treatment was in the use of contact lenses, with 14% of patients reporting their use as an initial treatment, and 39% reporting their use as a current treatment. Contact lenses were the third most common current treatment for oGVHD. The frequency of other treatments did not change substantially from initial to current use.

The most common therapeutic lens type used in the current study was scleral lenses. The scleral lens rests entirely on the conjunctiva and vaults over the cornea. The rigid structure of the scleral lens allows a layer of preservative-free saline to cover the surface of the eye. The large diameter of the lens over the conjunctiva also protects the surface of the eye from environmental exposure. Other studies have shown the benefit of scleral lenses for oGVHD treatment [25–27]. In a review of 33 patients with oGVHD, 97% of patients treated with scleral lenses experienced reduced eye pain and an improved quality of life [26]. Furthermore, 63% reported reduced or resolved light sensitivity. Patients in that study were treated with scleral lenses on the basis of dry eye symptoms and not the severity of signs.

Notably, midday fogging is a common issue among scleral lens wearers that may limit their success. Midday fogging refers to the blurred vision that some scleral lens wearers experience. In the current study, 84% of patients with oGVHD who wore scleral lenses reported midday fogging. In other reports of midday fogging for patients who wear scleral lenses for dry eye or for corneal irregularity, 26% to 46% of wearers had issues with midday fogging [28]. However, the causes and treatments of midday fogging remain elusive [18,29]. One study showed that redness and irritation occurred in patients who experienced midday fogging. Because oGVHD has an inflammatory component, the increased incidence of midday fogging reported by this group may be related to ocular inflammation in these patients. The possibility also remains that the midday fogging reported by these patients is due to poor surface wetting of the lens that often occurs in patients with meibomian gland dysfunction, which is a component of oGVHD. Although fewer patients with oGVHD in the current study wore soft lenses than scleral lenses, most of them (83%) also reported midday fogging with lens wear.

The burden of symptoms and treatments related to oGVHD can negatively affect patient quality of life [1,13,14,22]. The ocular surface is highly innervated; therefore, any disruption of the tear film or ocular surface structures can be exquisitely painful. Eye discomfort may be described as a burning, stinging, itching, or foreign body sensation; pain; conjunctival redness; or light sensitivity. Blurred or compromised vision can make common daily activities such as driving, computer work, being outdoors, or other social interactions difficult [30]. The goal of oGVHD treatment is to maintain ocular surface integrity by replenishing tear volume, slowing the drainage of existing tears, maintaining or improving meibomian gland function, and reducing ocular inflammation. Treatment typically consists of several discrete interventions, which must be carried out daily.

The burden of financial costs and time requirements associated with oGVHD may also negatively affect patient quality of life. In the current study, the yearly estimated median (IQR) out-of-pocket cost related to dry eye treatments for patients with oGVHD was USD 900 (USD 400–USD 2000), and the mean (SD) was USD 2618 (USD 7784). This is higher than the mean estimates previously reported for the general population, which were USD 783 per year in 2008 and USD 1294 per year in 2024 [31,32]. A 2023 study of patients with cancer showed that the mean out-of-pocket cost was USD 1058 higher for patients with cancer than for patients without cancer [33]. In a 2018 study, 23% of patients with GVHD spent more than USD 500 in 3 months on health care-related nonmedical costs, such as transportation to appointments [34]. The cost of care is magnified when considering that patients may lose earning potential with GHVD. A 2023 study found 67.9% of patients (*n* = 165) reported loss of income due to chronic GHVD, and of those, 52.2% reported they had lost more than 50% of their income [15]. In addition, dry eye treatment requires a time investment each day. In the current study, 39% of patients with oGVHD reported spending an hour or more every day on their dry eye treatment alone.

Patients with oGVHD reported frequent clinical visits for dry eye, with a mean (SD) of four (3) visits per year for dry eye care. Two patients reported no yearly visits for a dry eye evaluation, which may highlight a gap in care for these patients. A study in which patients were monitored after HSCT revealed that 46% (51/111) did not have an eye examination afterward [21]. Of those 51 patients, 11 had a high OSDI score, indicating symptomatic dry eye disease. Another study of eye examination referral rates among oncology patients who had undergone HSCT over a 20-month period revealed that only 29% had been referred to an ophthalmologist [35]. These referrals were made only when a patient reported ocular symptoms.

With the high prevalence of oGVHD after HSCT, referring patients for eye examinations is important to ensure the prompt diagnosis and treatment of dry eye disease. Patients should be encouraged to have eye examinations at regular intervals. In addition, they should be educated on the early signs of dry eye and urged to have an eye examination if symptoms occur. A 2012 study of screening practices after HSCT showed that eye examinations are recommended 6 and 12 months after HSCT and then yearly, or sooner, if any visual symptoms develop [36]. The American Academy of Ophthalmology recommends eye examinations every 3 to 12 months for patients who have undergone prior HSCT and are receiving systemic immunosuppressant therapy [37]. In a study of 161 patients after HSCT, slitlamp examination findings for dry eye and OSDI scores were evaluated, and it was suggested that the OSDI score may be a useful screening tool for patients to guide eye examination referrals, either in the clinic or at home [38]. Adding the 12-question OSDI questionnaire to regularly scheduled hematology examinations may be a simple way to screen patients for referral to an eye care specialist.

Although most patients with oGVHD in the current study reported receiving primary eye care from an eye care professional, 5% reported receiving treatment from their hematologist. Hematology teams have an important role in ensuring that patients with oGVHD are evaluated by other specialists when indicated. Hematologists should be able to recognize the symptoms of dry eye. In addition, they should have an established relationship with an eye care specialist who is knowledgeable about dry eye treatment, such as scleral lenses, to whom they can refer patients. An eye care professional skilled in dry eye disease can provide the most timely and effective treatments to manage the symptoms of dry eye. In the current study, only 40% of patients reported ease in finding a dry eye specialist.

Despite the fact that patients with oGVHD often undergo multiple treatments for dry eye disease, our study shows that they are likely to remain symptomatic and must balance daily treatments and frequent professional visits to manage their disease. As medical advancements continue to prolong the life expectancy of patients who undergo HSCT, health care professionals should strive to maximize their patients’ quality of life. This includes optimizing the treatment of ocular sequelae. Eye care professionals with experience and knowledge in the field of ocular surface disease should be included on each patient’s care team early in the course of care, preferably even before HSCT is performed. The prevention of ocular complications through timely and appropriate intervention may help minimize the risk of potentially sight-threatening issues. All health care professionals should be aware of symptoms that persist so that they can provide patients with the highest quality of life after HSCT.

This study had several limitations: First, the patient population was limited to those who participated in patient support groups and were willing and able to respond to an electronic questionnaire, which potentially introduced systematic bias. The initial study recruited any patient with dry eye and did not focus solely on oGVHD patients. Patient recall was relied upon for accuracy of self-diagnosis, treatments, and estimated values of money and time spent on dry eye treatment, all of which may not be accurate. Furthermore, our study was limited by the lack of clinical assessment of disease severity for these patients; therefore, all patient responses were subjective. Although initial and current treatments were recorded, reasons for changes in treatment were not ascertained.

Likewise, no assessment of current systemic health was available; thus, overall disease management may have contributed to ocular symptoms and their management. This study cannot represent the experience of all oGVHD patients but rather provides insight into a sample of patient-reported experience. A prospective multi-center study, following clinically diagnosed oGVHD patients, that includes clinical ocular exam findings, along with systemic and topical treatments, and assessment of patient quality of life and burden of care should be considered to more accurately address the unique needs of this dry eye population.

## 5. Conclusions

This study revealed that dry eye symptoms persist for many patients with oGVHD after HSCT, despite the use of multiple ocular therapies. While this study did not physically evaluate the patients’ ocular surface, it does highlight the importance of monitoring patient symptoms and treatment regimens. Furthermore, the associated cost and time patients devote to dry eye care may be burdensome. In the future, conducting a prospective study of consecutive patients with dry eye syndrome after allogeneic HSCT may provide additional insights into this condition and lead to improved care management strategies. Having access to a dedicated, multidisciplinary team trained to assess, intervene, and monitor ocular symptoms is critical for optimal outcomes and symptom management.

Patients with substantial ocular involvement in oGVHD benefit most from a multidisciplinary team composed of a transplant physician and an eye care professional with expertise in dry eye management. Together, they can assess the patient to determine optimal systemic and local therapy.

## Figures and Tables

**Table 1.** Patient summary.

Patient	Value
Age (mean ± SD)	55 ± 10 (range 22–73) years; *n* = 75
Years with dry eye (median, IQR)	4 (2–8); (range 0–31) years; *n* = 78
OSDI score (mean ± SD)	56 ± 23 (range 10–100); *n* = 79
Number of symptoms (mean ± SD)	9 ± 2 (range 0–11); *n* = 79
Number of treatments (mean ± SD)	4 ± 2 (range 1–9); *n* = 79

**Table 2.** Patient-reported symptoms.

Symptom	Value^a^ (*N* = 79)
Sandy/gritty feeling	68 (86)
Blurry or fluctuating vision	64 (81)
Sensitivity to light	63 (80)
Tired or fatigued eyes	59 (75)
Eye pain, discomfort, or ache	56 (71)
Burning or stinging eyes	52 (66)
Red eyes	44 (56)
Itching	39 (49)
Swollen or red eyelids	32 (41)
Sticky tears	25 (32)
Watering or tearing	20 (25)

a Values are reported as No. (percentage).

**Table 3.** Initial and current patient-reported treatments.

Treatment	Timing of treatment	Value ^a^ (*N* = 79)	Percent change
Artificial tears	Initial	71 (90)	−3
	Current	69 (87)	
Warm compress	Initial	41 (52)	+1
	Current	42 (53)	
Punctal plug or cautery	Initial	37 (47)	−9
	Current	30 (38)	
Cyclosporine drop	Initial	35 (44)	−22
	Current	17 (22)	
Artificial tear gel	Initial	29 (37)	−8
	Current	23 (29)	
Artificial tear ointment	Initial	25 (32)	−12
	Current	16 (20)	
Steroid drop	Initial	24 (30)	−5
	Current	20 (25)	
Lid cleaner	Initial	12 (15)	+4
	Current	15 (19)	
Autologous serum drops	Initial	12 (15)	+12
	Current	17 (22)	
Contact lens	Initial	11 (14)	+25
	Current	31 (39)	
Antibiotic drop	Initial	8 (10)	−1
	Current	7 (9)	
Oral antibiotic	Initial	8 (10)	−4
	Current	5 (6)	
Moisture chamber glasses or goggles	Initial	6 (8)	+8
	Current	13 (16)	
Allergy drop	Initial	5 (6)	−2
	Current	3 (4)	
Lifitegrast drop	Initial	2 (3)	−2
	Current	1 (1)	
Meibomian gland expression	Initial	2 (3)	−3
	Current	0	

a Values are reported as No. (percentage).

## Data Availability

The data supporting the findings of this publication can be made available upon request
